# Research progress of endometrial receptivity in patients with polycystic ovary syndrome: a systematic review

**DOI:** 10.1186/s12958-021-00802-4

**Published:** 2021-08-06

**Authors:** Xuechun Bai, Lianwen Zheng, Dandan Li, Ying Xu

**Affiliations:** The Second Hospital of Jilin University, Jilin Province Changchun City, China

**Keywords:** Endometrium, Polycystic Ovary Syndrome, Biomarkers, Metabolism, Embryo Implantation

## Abstract

Polycystic ovary syndrome (PCOS) is a neuroendocrine heterogeneous disease that frequently occurs in women of reproductive age, causing serious damage to the fertility, quality of life, and physical and mental health of patients. The current studies have proved that satisfactory endometrial receptivity is one of the conditions that must be met during the process of spermatovum position, adhesion and invasion, as well as the subsequent blastocyst division and embryo development. Women with PCOS may suffer a series of pathological processes such as changes in the expression levels of hormones and related receptors, imbalances in the proportion of miscellaneous cytokines, insulin resistance, low-grade chronic inflammation and endometrial morphological changes, which will damage endometrial receptivity from various aspects and obstruct fertilized egg nidation and embryonic development, thus causing adverse reproductive health events including infertility and abortion. This article reviews the research progress about characteristics and related influencing factors of endometrial receptivity in PCOS patients.

## Background

Polycystic ovary syndrome (PCOS) is a neuroendocrine heterogeneous disease that commonly occurs in women of childbearing age and is one of the major factors of female infertility. The clinical manifestations of PCOS mainly include amenorrhea, infertility, hirsutism, acne, and obesity. It is characterized by chronic persistent ovulatory dysfunction, abnormally elevated levels of serum androgen, and changes in polycystic ovary morphology, usually associated with a pathological condition, such as obesity, insulin resistance (IR), and low-grade chronic inflammation [5, 72]. The prevalence of PCOS varies with diagnostic criteria, study area, and race. Its prevalence was 8.7% ± 2.0% under the National Institutes of Health (NIH) standard diagnosis. The prevalence was 11.9% ± 2.4% according to the Rotterdam criteria and 10.2% ± 2.2% when assessed using the American Androgen Excess Society (AES) recommendations [57]. Studies have clearly shown that the incidence of PCOS has reached 5.9%–6.8% in Asian women aged 15–39 y [105] and shown an increasing trend. The proposal of PCOS dates back to 1935. After more than 80 years of research, its pathogenesis remains inconclusive. PCOS certainly not only affects ovulation and the normal menstrual cycle in women of childbearing age but may also increase the risk of endometrial cancer, type II diabetes, and cardiovascular disease [9, 35, 104], causing serious damage to the fertility, quality of life, and physical and mental health of patients. PCOS is a common conundrum in obstetrics and gynecology, reproductive medicine, and endocrinology and metabolism.

Endometrial receptivity refers to a series of physiological changes in which the endometrium provides the best environment for embryo localization, adhesion, invasion, and implantation. Notably, whether its timing and degree of tolerance exhibit synchronous alteration with the growth of the fertilized ovum is key to determining whether the latter can be successfully implanted [43]. The endometrium generally shows maximum receptivity 6–9 d post-ovulation, a period referred to as the implantation window that typically lasts 30–36 h [80]. It requires the support of estrogen and progesterone secreted by the corpus luteum, and is also affected by various genes, proteins, cytokines and adhesion molecules. This period is crucial for blastocyst adhesion and implantation into the receptive endometrium. Meanwhile, the invasive ability of the embryo perfectly matches the receptivity of the endometrium, after which a close "dialogue" mechanism can be established to complete the implantation [66]. If implantation is outside the range of implantation window, the development of the endometrium and the embryo is not synchronous. Thus, the condition of the endometrial material and acceptability fail to meet the requirements for fertilized egg implantation, impeding the nidification of embryos into the endometrium [43, 101].

Compared with normal individuals, PCOS patients have a series of adverse pregnancy outcomes, such as low embryo implantation rate and high abortion rate. Moreover, among the multitudinous factors and mechanisms leading to these results may be the reduction of uterine endometrial receptivity. In recent years, the mechanism of impaired endometrial receptivity in PCOS patients has drawn research interest in reproduction. This article briefly reviews the progress of current research on the characteristics and related influencing factors for endometrial receptivity in PCOS patients.

## Main text

### Diagnosis and classification of PCOS

The pathogenic factors of PCOS are currently undetermined. The incidences vary in disparate ethnic groups, various living environments, and diverse behavioral habits. The clinical manifestations are also highly heterogeneous, and the lesions involve various systems. Therefore, the diagnostic criteria have not been standardized. The major international diagnostic criteria currently proposed include the NIH standard by the NIH, the Rotterdam criteria suggested by the European Society for Human Reproduction and Embryology/American Society for Reproductive Medicine, and the AES criteria advanced by AES [6]. At present, the Rotterdam criteria are used as the diagnostic criteria for PCOS in most countries and regions.

The Rotterdam criteria are as follows: (1) Biochemical manifestations of hyperandrogenism or/and clinical manifestations of hyperandrogenism; (2) Anovulation or rare ovulation; (3) Ultrasound indicates that ≥ 12 follicles with a diameter of 2–9 mm are found on the same surface of the ovary, or the ovarian volume exceeds 10 mL. PCOS can be diagnosed if it meets two of the three aforementioned points (Rotterdam 2004 [78]). It is necessary to exclude other diseases that may cause hyperandrogenism and those that may cause ovulatory dysfunction, such as thyroid diseases, hyperprolactinemia, androgen-secreting tumors, Cushing's syndrome, and delayed congenital adrenal hyperplasia, which may cause similar symptoms(Endocrinology Subgroup and Expert Panel 2018 [20]).

#### Diagnostic basis of PCOS

Medical history(Gynecologists 2018 [27]): (1) Presence of symptoms, such as acne, alopecia, seborrheic dermatitis, and acanthosis nigricans. (2) General information, including age, height, weight, body mass index, waist circumference, hip circumference, and so on. (3) Menstrual history and marriage and pregnancy history: age at menarche, menstrual cycle, menstrual time, changes in menstrual volume, duration of infertility, adverse pregnancy history (e.g., spontaneous abortion or embryo arrest). (4) Family history of diabetes, hypertension, obesity, and PCOS (specifically in parents and first-degree relatives of PCOS patients). (5) Lifestyle, including diet, exercise, smoking history, and alcoholism history.

Laboratory diagnosis(Gynecologists 2018, [97]: (1) The luteinizing hormone/follicle-stimulating hormone (LH/FSH) ratio was disproportionate—for instance, LH/FSH > 2. (2) The estrone/estradiol (E1/E2) ratio was not well-proportioned, and serum estrogen was mostly normal or slightly increased without periodic changes. (3) The androsterone and serum testosterone concentrations were elevated (generally not more than twice the upper limit of the normal range). (4) The fasting blood glucose level, 2–hour postprandial blood glucose level, fasting insulin levels, blood insulin levels after glucose administration, and insulin area under the curve are usually increased. (5) The level of anti-Müllerian hormone (AMH) is significantly increased; meanwhile, no precise cut-off value or distinct individual and ethnic differences in AMH are currently determined. (6) Triacylglycerol, total cholesterol, and low-density lipoprotein cholesterol are typically increased. (7) Other exclusionary laboratory measurements include thyroid function tests, cortisol concentrations, corticotropin-releasing hormone, and the 17-hydroxyprogesterone test, among others.

Imaging diagnosis(Gynecologists 2018, [42]: (1) Ultrasound: Sex hormone drugs should be discontinued for at least 1 month before ultrasonography. On the basis of ensuring the absence of the corpus luteum, a cyst, or a dominant follicle, increased ovarian volume can be seen on ultrasound (ovarian volume = 0.5 × long diameter × transverse diameter × anteroposterior diameter). The capsule and interstitium of the ovary were proliferated; simultaneously, the echo was enhanced, and the contour was smooth. More than 12 anechoic areas (2–9 mm in diameter) were present on one or both sides of the ovary surrounding the ovarian margin. Moreover, continuous monitoring revealed no signs of ovulation during a certain period. Notably, the morphology and function of the ovary as a woman ages are metabolic. In adolescent women, the volume of the ovary is small, and the hypothalamus–pituitary–ovarian axis feedback and function are not perfect; in perimenopausal women, the volume is decreased, and the function may decline. Therefore, the diagnosis of PCOS should have different thresholds in women of different ages. Some studies suggest lowering the diagnostic threshold of the ovarian volume and the antral follicle count from the age of 30 [38]. (2) Magnetic resonance imaging: image characteristics include increased ovarian volume, increased antral follicle count, abnormal distribution of peripheral follicles, and increased central stroma [7].

In short, the diagnosis of PCOS should be comprehensively evaluated according to the clinical symptoms, changes in hormonal levels and metabolites, and imaging test results of the patient.

#### Classification of PCOS

The Evidence-based Methodology Workshop on PCOS in 2012 held by NIH proposed the phenotype specification, which classified PCOS patients into 4 subtypes based on different diagnostic criteria met by different clinical manifestations, laboratory, and imaging findings: (1) Phenotype A (classical PCOS), which satisfies the three diagnostic criteria in Rotterdam. (2) Phenotype B (hyperandrogenic and anovulatory PCOS), which satisfies two diagnostic conditions of hyperandrogenism and/or hyperandrogenism and rare ovulation or anovulation. (3) Phenotype C (ovulatory PCOS) in which clinical and/or biochemical manifestations of hyperandrogenism and ovarian morphology are detected on ultrasound. (4) Phenotype D (non-hyperandrogenic PCOS), which shows rare ovulation or anovulation in patients and polycystic ovarian changes, are observed on ultrasound.

The distribution of PCOS subtypes largely varies among different ethnic and regional populations. In European and American populations, Type A is the most commonly occurring type, followed by Type C or D, and ultimately by Type B [77]. in the Chinese population, type C comprises the highest proportion, followed by types A, D, and B [109]. Studies have shown that the characteristics of metabolic abnormalities vary in different types of PCOS. Types A and B, characterized by abnormal hormone levels, hyperandrogenism, IR, and abnormal lipid metabolism, are more severe than other types [99]. Their clinical manifestations are also more easily recognized, such as irregular menstruation, hirsutism of the skin, multiple acne on the face, and acanthosis nigricans [26, 32]. By contrast, several reports contradict the conclusion that phenotypes A and B comprise about two-thirds of the total number of PCOS patients identified in current clinical settings. These data suggest that about two-thirds of PCOS patients identified in unselected populations can be classified into phenotypes B and C; meanwhile, phenotypes A and D are almost equally prevalent [41]. Other studies demonstrate no significant differences in clinical manifestations between various types of PCOS [85].

The classification of PCOS remains inconclusive, and its diagnostic criteria have not been standardized. In the current study, the clinical classification of women with clinically confirmed PCOS was yet to be clarified, although few data were presented on identification classification in the unselected population; thus, the changes in endometrial receptivity in a certain type of patients were studied separately. Thus, this study discusses the characteristics and the factors influencing endometrial receptivity in PCOS patients in general.

## Characteristics of endometrial receptivity markers in PCOS patients during the implantation window

### Characteristics of endometrial morphology markers during the implantation window.

#### Physiological state

**Fig. 1:**
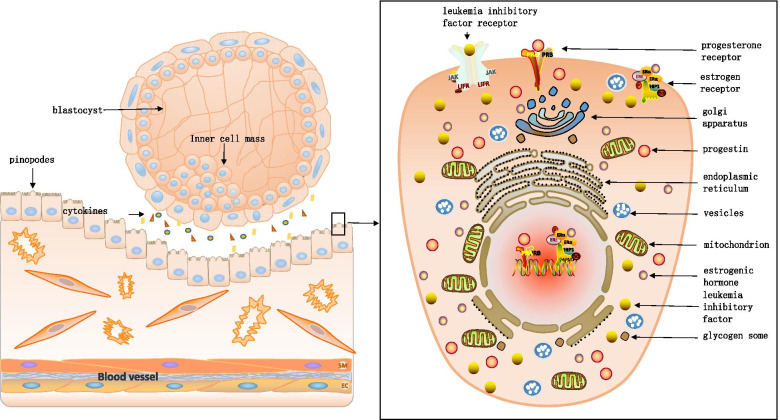
Structure chart of pinopodes

During the implantation window, a membranous bulge is formed by the fusion of microvilli at the top of endometrial epithelial cells covered by the endometrial layer of the uterine cavity as observed the scanning electron microscope. This bulge consists of pinopodes (pps) [69]. The growth of the bulge can be divided into three stages: (1) germination and development, (2) gradual maturation, and (3) atrophy and degeneration [4]. The rough endoplasmic reticulum, Golgi apparatus, mitochondria, and other organelles, in addition to vesicles, glycogen granules, and other substances, are found in the pinopodes. Its exterior has diversified receptors, such as the leukemia inhibitory factor (LIF) receptor, estrogen receptor (ER), and progesterone receptor (PR) [59]. which mediates the absorption of fluid and certain nutrients or cytokines from the uterine cavity by endometrial cells (Fig. 1). The AQP gene may also involve the regulation of the pps function. Studies have clearly indicated that the deletion of the AQP2 gene can reduce the ability of pps to cling to endometrial cells [29]. These structures on the surfaces of pps are necessary for the blastocyst to adhere to the endometrium. Thus, the structural integrity of pps is closely related to the implantation outcome [95]. The occurrence and development of the pps structure are hormone-dependent. Its formation and degeneration are regulated by the concentration of progesterone (P), the density of PR, and the secretion level of estrogen (E). When the concentration of P and density of PR in the body increase, the number of pps increases, and the structure becomes more intact, whereas the level of E is closely related to the degeneration of pps. Moreover, the development and maturation of pps exhibit synchronous changes with the opening of the endometrial implantation window [70]. On Day 4 of pregnancy, the pps in the endometrial epithelial cells of infertile patients treated with gonadotropin-releasing hormone agonist (GnRH-α) were significantly larger in number and more mature than those of patients without treatment. The clinical pregnancy rate of the treated patients was also improved, suggesting that the number and maturity of pps can reflect the degree of endometrial receptivity and are positively correlated with the embryo implantation rate [111]. Thus, during the opening of the implantation window, the number and developmental status of pps more accurately reflect the status of endometrial receptivity, which can be used as a distinct morphological marker of the latter [73].

The nucleolar channel system (NCS) is another morphological marker of endometrial receptivity, which transiently resides in the glandular epithelial cells of the human midluteal endometrium and is evenly distributed in the superficial cells of the endometrial cavity [83]. The NCS refers to a type of miniature organelles, consisting of several layers of membrane tubules embedded in an electron-dense matrix [60]. The NCS is surrounded by an electron-dense matrix, localized within the nucleus without membranes. The formation threshold of the NCS is determined by the lowest concentration of P and almost overlaps with the minimum value of P (3 ng/mL) required for ovulation [61]. Other studies have confirmed that the appearance of NCS is almost consistent with the implantation period of the endometrium. The former regulates the implantation process by influencing the synchronous development of the zygote and the endometrium, indicating that it plays a certain role in the initiation and maintenance of endometrial receptivity [81].

#### PCOS patients

Experimental results have demonstrated that PCOS patients have poor maturation of pps and wretched endometrial typing, indicating that its downregulation and cacoethic structural development are among the factors triggering the reduction in the endometrial receptivity of PCOS patients during the implantation window [34]. A trial of intrauterine instillation of human chorionic gonadotropin (hCG) in infertile PCOS patients indicated no significant difference in serum E_2_ levels between the control group and the perfusion group during the implantation window; however, the serum P level was markedly higher in the perfusion group than in the control group during the implantation window. Moreover, the number of mature pps in the endometrium during the perfusion cycle was considerably greater than that in the control group. This difference suggests that the pps exhibited a hypoplastic structure, the number of mature pps was reduced, and endometrial receptivity was damaged during the implantation window in PCOS patients; meanwhile, hCG promoted the formation and development of pps. The uterine cavity perfusion of hCG would also be capable of increasing the number of mature pps and improve endometrial receptivity [92].

Compared with the control group, PCOS patients exhibited lower NCS expression in the endometrial epithelium in the midluteal phase. The endometrial classification was also inferior, as reflected by small endometrial glands and a narrow glandular lumen. This characteristic may have also led to impaired endometrial receptivity. Moreover, the proportion of the endometrium in the middle secretory phase of the control group to that of the normal female group was 33.33%: 73.33%. The endometrium of the control group was mostly in the proliferative and early secretory phases, with coarse endometrial receptivity. This feature is not conducive to the implantation of fertilized eggs, resulting in a decline in the pregnancy rate [33]. Analysis of exfoliated cells in aspirated endometrial secretions via indirect immunofluorescence techniques for NCS reveals a higher NCS detection rate in endometrial epithelial cells from successful pregnant patients, suggesting that NCS expression can be used to determine optimal endometrial acceptance and improve pregnancy outcomes [82].

### Characteristics of endometrial molecular biological markers in PCOS patients during the implantation window

#### Physiological state

In certain pathological processes, the following can affect the endometrial environment: abnormal development of fertilized eggs; the excessive immune response of the body; embryonic wound repair; malignant tumor metastasis; expression, time, and intensity of genes (e.g., homeobox gene-A10, homeobox gene-A11, SRY-associated HMG box gene 17, etc.); proteins (e.g., estrogen receptor, progesterone receptor, androgen receptor, integrin, osteopontin, type IV collagen, pregnancy-associated protein, etc.) expressed by endometrial tissue cells during the implantation window; and many cytokines (such as the LIF, interleukin, colony-stimulating factor, and vascular endothelial growth factor, among others) induced for secretion. These factors contribute to changes in the initiation time and persistence of endometrial receptivity, leading to adverse pregnancy events such as infertility and abortion [37, 62, 84].

Currently, the most widely studied related genes are homeobox gene-A10(HOXA-10) and homeobox gene-A11(HOXA-11). HOXA-10 is a key gene influencing embryo implantation governing the expression of decidual markers, namely, insulin-like growth factor binding protein-1 (IGFBP-1). HOXA-11 typically inhibits the prolactin gene [58]. The HOXA-10 gene also often affects the establishment of endometrial receptivity by binding to MEIS1, a transcription factor of one of the TALE homology family members, to form a heterodimer. The specificity of its own binding to downstream target genes is thus enhanced, strengthening the regulation of its function. MEIS1 is expressed in endometrial stromal cells and glandular epithelial cells throughout the menstrual cycle; nonetheless, its expression is significantly higher in the midsecretory phase than in other periods, which may be attributed to the appearance of the implantation window [12].

MicroRNAs (miRNAs), non-coding RNAs with highly conserved expression, are usually composed of 18–22 nucleotides. They regulate gene expression at the post-transcriptional level by targeting mRNAs for cleavage or transcriptional repression.

The main mechanisms of the LIF during blastocyst implantation mainly include the following: (1) triggering the decidual response of the endometrium, which is conducive to embryo invasion; (2) enhancing the developmental ability of endometrial stromal cells and protecting stromal cells from damage during embryo implantation and trophoblastic invasion; (3) altering matrix metalloproteinase (MMPs) and the mRNA expression of the urokinase-type proenzyme activator, indirectly enhancing the invasion of trophoblastic cells; (4) stimulating trophoblastic cells to secrete fibronectin and promoting the expression of endometrial integrin αvβ3, accordingly facilitating adhesion indirectly.

Interleukin-6 (IL-6) is a cytokine produced during a series of immunological processes, such as pro-inflammation and anti-inflammation induced by maternal pregnancy. Its indirectly bear upon endometrial receptivity by transforming the level of E_2_ and performs its functions in blastocyst formation and embryo implantation [76].

MMPs represent a class of protein-interrelated hydrolases that degrade the extracellular matrix and allow the smooth implantation of the blastocyst into the endometrium. MMPs play a major role in the cyclical changes in the endometrium, embryo implantation, and embedding [15], Zhang et al. 2007 [110]). MMP-2 and MMP-9 promote follicular development, as well as play an indispensable role in the selection of dominant follicles [86]. MMP-2 can further facilitate trophoblastic infiltration and embryo implantation [11].

#### PCOS patients

Changes in endometrial receptivity depend on the spatiotemporal specific expression of certain particular genomes in the endometrium. Compared with those in the normal group and obese ovulatory group, the gene spectrum in patients in the PCOS group changed significantly, and 12 of the 25 genes selected and tested were abnormally expressed. The majority of these 12 genes regulated the cell proliferation, differentiation, and apoptosis of genes, such as GPX3, PAEP (a glycoprotein), and LIF*.* These genes can transcribe and translate related cellular molecules to modulate the basic metabolic processes and normal biological functions of the endometrium. These cytokines interact with their environmental factors and hinder embryo implantation by influencing the acceptance process of the endometrium [10].

Sampling and analysis of the endometria of PCOS patients indicate that the expression of HOXA-10 and HOXA-11 genes is reduced, and endometrial decidualization is prevented. Consequently, endometrial receptivity is impaired, which can lead to infertility in women with PCOS [36]. Meanwhile, PCOS patients exhibit progesterone resistance, thereby reducing the expression of progesterone-dependent HOXA-10 and HOXA-11. Studies have shown that progesterone resistance in PCOS patients undergoing laparoscopic ovarian drilling (LOD) is improved, and the mRNA expression of HOXA-10 and HOXA-11 is significantly increased. The expression of HOXA-10 in the midluteal phase is essential for blastocyst implantation, which improves endometrial acceptance [88].

Widely occurring in cells and tissues, miRNAs participate in multiple biological processes, including those highly correlated with reproductive disorders. A study analyzed the expression profile of miRNAs in the endometrium during the implantation window by using a gene chip; miRNA-223 was found to exhibit markedly reduced expression and could directly act on LIF and downregulate its protein expression. Consequently, the formation of pps in endometrial epithelial cells was disrupted, and endometrial receptivity was impaired, inhibiting embryo implantation [30, 89]. Meanwhile, miRNA-449a is the most visibly differentially expressed miRNA on the endometrium during the window stage, which can negatively adjust the transcription and translation of G protein-coupled receptor 4 (LGR4). LGR4 remains with the GPCR family, also known as GPR48, which contains leucine-rich repeats and plays a crucial role in the development of the reproductive system, endometrial decidualization, embryo implantation, and other life activities. Therefore, miRNA-449a has the potential to indirectly influence endometrial receptivity by lowering LGR4 expression [54]. Studies have demonstrated that miRNA-27a expression in PCOS patients is higher than that in the control group and is closely related to clinical features such as hyperandrogenism and IR. This finding suggests that there is expression of miRNA-27a in the occurrence and development of PCOS. Thus, it can be used as a potential indicator for the diagnosis and monitoring of PCOS progression [51]. Researchers have also recently found that certain miRNAs partly modulate the adhesive properties of endometrial epithelial cells. Moreover, noncoding long-stranded RNA secreted by embryos coordinates miRNAs to produce signals linking the maternal endometrium and the embryo. Meanwhile, the process by which miRNAs affect endometrial receptivity and the mechanism of interaction between the endometrium and the embryo remain inconclusive [21].

**Fig. 2:**
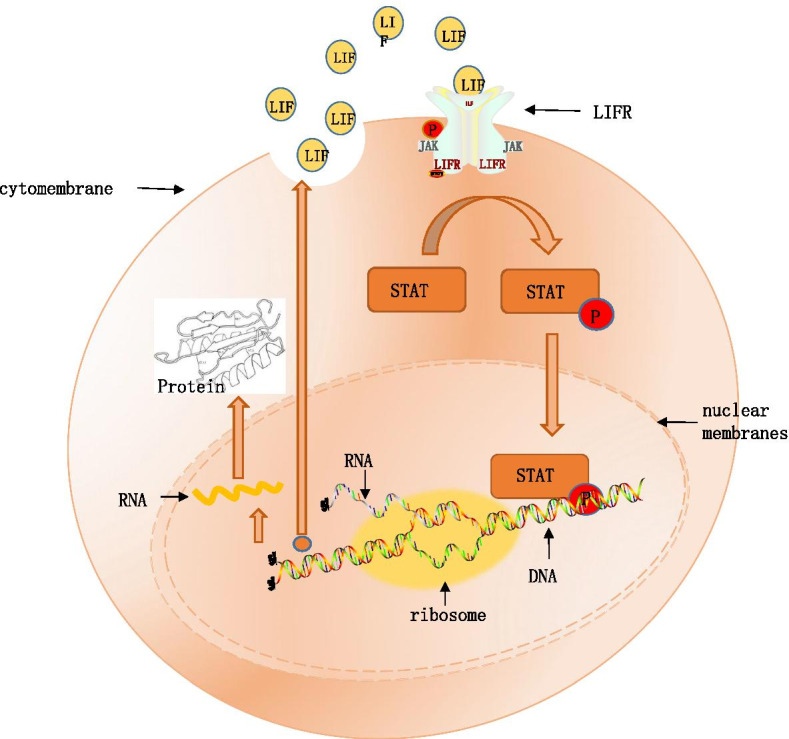
Signaling pathways of interleukin

LIF activates intracellular JAK and STAT pathways [58] (Fig. 2), and the downstream proteins of these two pathways are essential proteins for blastocyst implantation. A series of studies have distinctly assumed that LIF secretion levels are decreased in PCOS patients [67]. The consequences of the aforementioned changes, reduced expression of downstream proteins leads to impaired endometrial receptivity and causes implantation failure. Surveys have indicated that the combination therapy of pregnant mare serum gonadotropins and a gonadotropin-releasing hormone (GnRH) agonist can enhance the expression of integrin b3 and LIF and boost engraftment. This protocol is expected to improve uterine receptivity by restoring physiological endometrial secretion during ovarian stimulation cycles [25]. In a mouse model of PCOS, an impaired LIF-STAT3 pathway could be detected, and complex endocrine system dysregulation may cause decreased expression levels of LIF. Thus, low LIF levels adversely affect endometrial acceptance, eliciting a descendent embryo implantation rate [44].

IL-6 expression was found to increase in a mouse model of PCOS. It mediates chronic low-grade inflammation of the endometrium and produces various metabolic disorders, thereby reducing endometrial receptivity [48].

A domestic study found that the expression levels of MMP-9 and MMP-2 were significantly lower in the endometria of PCOS patients with ovulatory dysfunction (P < 0.01) than in those of the control group. The mechanism may be the immature follicles in PCOS patients and the disturbed endocrine level in the body. Notably, the opening of the endometrial implantation window and the development of blastocysts are not synchronous. Consequently, blastocysts are difficult to implant, ultimately causing infertility in patients(Zhang et al. 2007).

The leptin content in serum also influences the extent of the opening of endometrial receptivity. When leptin secretion in the body is considerably increased in vivo, endometrial tolerance can be reduced by prompting the STAT3 signaling pathway and downregulating γ-ENaC expression in the endometrium. Compared with the controls, PCOS patients with a high body mass index express more leptin, impairing their endometrial receptivity and lowering the pregnancy rate [49].

3.Related influencing factors for endometrial receptivity in PCOS patients during the implantation window.

### Effect of sex hormones and their receptors on endometrial receptivity in PCOS patients(Fig. 3)

**Fig. 3:**
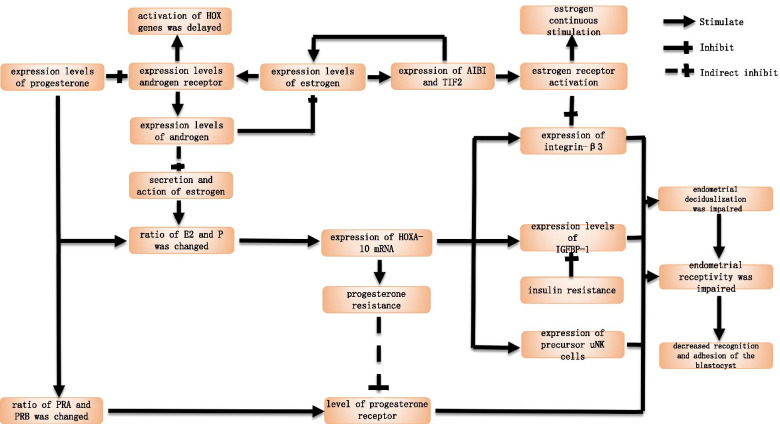
How sex hormones affect endometrial receptivity

#### Physiological state

E acts a primary role in the initial differentiation of the endometrium, and P promotes the transformation of the endometrium from proliferative to secretory phases, rendering the endometrium suitable for embryo implantation and growth. Moreover, E and P can adjust HOXA-10 expression. Only when the levels of the E and P in vivo reach equilibrium, can the expression of HOXA-10 mRNA reach a maximum, rendering endometrial development more appropriate for embryo implantation [40, 74]. Meanwhile, androgen decreases endometrial receptivity by inhibiting the regulation of E on HOX genes, contributing to the blockage of blastocyst implantation and embryonic development.

Endometrial receptivity and E are normally established simultaneously, while the ability of the endometrium to respond to E depends on the ER density on the cell surface. ER includes two structures, ERα and ERβ, and their expression levels in endometrial glands and stroma exhibit periodic variation. During the proliferative phase, the endometrium is continuously stimulated by higher levels of E, facilitating the manifestation of ER. Therefore, ER has the highest content in the late proliferative phase and is significantly reduced with a decrease in E after ovulation.

Among the multiple factors affecting embryo implantation, the key link identified is the increase in P in the secretory phase and the downregulation of PR. During the ovulatory period, incremental expression of ERα would irritates levels of E_2_ and indirectly accelerates PR expression. Although the content of the latter is gradually decreased in glandular epithelial cells, its content is relatively increased in mesenchymal cells, reflecting a corresponding increase in the total amount.

The androgen receptor (AR), which is also found in human endometrial glandular epithelial cells and stromal cells, mediates the vital role of androgens in target cells in vivo. Its activation is initiated by AR binding with androgens such as testosterone (T) and dihydrotestosterone.

#### PCOS patients

Some studies have shown that considerably high or low levels of E and P and the value of E_2_/P are unfavorable for the synchronous process of endometrial tolerance, blastocyst implantation. and implant window opening [39]. In PCOS patients, elevated androgen concentrations in the follicular phase may prevent or delay the timing of HOX gene activation [13], which may lead to weak endometrial receptivity in PCOS patients with hyperandrogenism.

Owing to the presence of a persistent ovulatory disorder in PCOS patients, luteal development is impaired, resulting in a decline in P relative to that in non-PCOS patients. The endometrium is stabbed by E without P resistance for a long time. After PCOS patients enter the secretory phase, the expression of the ER activator AIBI and the transcription mediator TIF2 in the endometrium is markedly increased, further activating ERα, which upregulates E in patients and enhances the long-term stimulation effect of E. The vicious cycle of the two affects the establishment of benign endometrial receptivity [67]. In addition, when the expression of ERα increases, the expression of integrin β3 decreases. Meanwhile, integrin can mediate bidirectional recognition, adhesion, and blastocyst implantation into the endometrium, Consequently, the decrease in its expression engenders maternal recognition and adhesion dysfunction of the blastocyst, which is one of the important factors for the breakdown of endometrial receptivity [100].

Two isoforms of PR exist in the endometrium: PRA and PRB. In the cyclical changes in the endometrium, the expression levels of both fluctuate in an isotype and cell-specific manner, and their ratio affects the endometrial response to progesterone and the magnitude of action [46]. However satisfactory endometrial tolerance is inextricably linked to the role of P. Therefore, the reduced PR expression or imbalance of disparate isoforms negatively affect endometrial receptivity. During the implantation period, abnormally high PR expression indicates progesterone resistance [18]. and can prompt the occurrence of adverse outcomes, such as decreased luteal function, infertility, and miscarriage. Proximate investigations have indicated that the mRNA and protein expression of PRA in PCOS patient groups are higher than those in non-PCOS patient groups, indicating the presence of progesterone resistance in the PCOS population. This factor may be one of the causes of impaired endometrial receptivity in PCOS patients [31].

Overexpression of the components of MAGEA11 that enhance AR expression can be observed throughout the menstrual cycle in PCOS patients. A molecular complex is formed and co-localized in stromal tissues and nuclei in PCOS patients. Their mediated transcriptional regulation may influence the decidualization response of the endometrium, which then disrupts endometrial receptivity in these infertile women [106]. AR within endometrial epithelial cells is generally regulated by ovarian hormones. A number of studies hypothesize that E_2_ can upregulate, whereas P and epidermal growth factor can downregulate AR expression [23]. Thus, the highest AR expression is observed in the proliferative endometrium stage and is gradually decreased in the secretory phase. Endometrial epithelial AR expression levels are markedly increased in PCOS patients relative to those in normal controls. This observation further amplifies the adverse effects of androgens on endometrial development and embryo implantation, consequently disrupting endometrial receptivity in PCOS patients [47, 71].

### Effect of low-grade chronic inflammation on endometrial receptivity in PCOS patients

#### Physiological state

Similar to allogeneic transplantation, embryo implantation is a complex course involving numerous immunomodulatory factors, such as the immunomodulation of uterine natural killer cells (uNK cells) and the balance of Th1/Th2 cytokines. A normal female endometrium contains dozens of immune cells, specific cytokines, and chemokines, which perform their essential roles in normal endometrial function [17]. The secretion of these inflammatory factors and immune cells is controlled by the pulsatile release of sex hormones, characterizing periodic changes. In the follicular phase, T cells are predominant as the content of secretory endometrial uNK cells increases. Studies have confirmed a significant positive correlation exists between the degree of maturation of endometrial tissue and the number of uNK cells [98]. These cells increase rapidly in the middle and late stages of the menstrual cycle, are mainly distributed in the decidua basalis, and frequently interact with the embryonic extravillous trophoblast (EVT) of the embryo. The critical role of uNK cells is to maintain the equilibrium of Th1/Th2 cytokines in the local immune response of the endometrium. Th1 is a cytokine mediating immune rejection at the maternal–fetal interface, which exhibits strong cytotoxicity, and Th2 is involved in immune tolerance. During the implantation period, high levels of progesterone prompt endometrial decidualization, followed by remodeling of the uterine spiral artery. EVT ultimately invades the maternal decidua and forms an immune microcirculation unique to the maternal–fetal interface with decidual immune cells and decidual stromal cells, which is key to successful embryo implantation [24, 75].

#### PCOS patients

PCOS is currently regarded as a low-grade chronic inflammation, which can induce several metabolic disorders and long-term complications. Women with PCOS ordinarily exhibit oxidative stress and elevated CRP relative to those of normal women [8]. A large volume of reports have substantiated that not only inflammatory factors such as IL-6 and CCL2 are increased in the proliferative endometrium of PCOS patients; the number of uNK cells in the endometrium is also distinctly reduced in the late secretory phase. All aforementioned can destroy the normal immune system of the endometrium, and the latter may be one of the crucial factors harming endometrial receptivity in PCOS patients ( [1, 79]. Surveys have verified that if uNK cells are activated owing to the secretion of certain inflammatory factors before or after embryo implantation, they can induce an increase in Th1 cytokines and a relative reduction in Th2 in vivo. The imbalance between the two can lead to a loss of endometrial receptivity and failure of embryo implantation [55]. After injection of human umbilical cord mesenchymal stem cells in mice with premature ovarian failure, the serum levels of E_2_, P, and IL-4 were markedly increased. The number of uNK cells and the ratio of Th1/Th2 cytokine in mice were measured by flow cytometry and immunohistochemistry, which showed evident reductions in both. The endometrial immune environment was simultaneously improved, allowing the recovery of endometrial receptivity in mice to a certain extent. This result indicates that the latter is regulated by the number of uNK cells and the ratio of Th1/Th2 cytokines [55]. Moreover, compared with those in normal women, the local microenvironment in which endometrial epithelial cells are located is altered in PCOS patients. For instance, CD68^+^ macrophages, CD163^+^ M2 macrophages, CD1a^+^ immature dendritic cells, CD83^+^ mature dendritic cells and CD8^+^ T cells are evident. Moreover, the disequilibrium in the proportion of various immune cytokines in the immune system and the formation of pro-inflammatory environment damage endometrial cells and hinder their proliferation and differentiation, resulting in unhealthy endometrial receptivity and poor embryo implantation. Such obstructions lead to infertility, abortion, premature delivery, and other adverse pregnancy outcomes [53]. A controlled clinical trial concluded that the persistent pregnancy rate of patients treated with GnRH-α was 42.0%, whereas that of the control group was 18.0% (*P* = 0.001). Notably, the abortion rate in the intervention group was 2.6%. It is considerably lower than that of the control group, which was 33.3% (*P* = 0.001). The use of GnRH-α intervention was demonstrated to affect the endometrial microenvironment, improve the inflammatory status, and enhance the endometrial receptivity and effect of embryo implantation, which can help maintain the normal pregnancy process and reduce the abortion rate in PCOS patients [2].

**Fig. 4:**
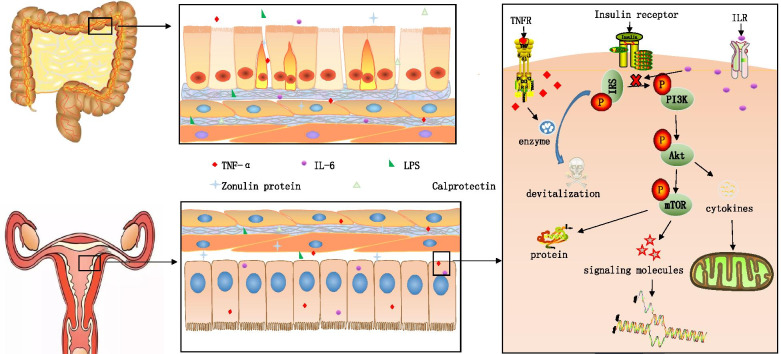
Intestinal barrier-endotoxemia-inflammatory mechanism

Some studies have proposed that the occurrence of PCOS is closely related to the hypothesis of “intestinal barrier–endotoxemia–inflammatory mechanism” (Fig. 4). PCOS patients exhibit reduced intestinal barrier function relative to that of the control group. A string of cellular molecules, signaling molecules, or inflammatory factors produced by intestinal bacteria, such as zonulin, calcium defense protein, lipopolysaccharides, tumor necrosis factor-α (TNF-α), and IL-6 can enter the blood via the damaged intestinal epithelial cell gap, causing endotoxemia and triggering an inflammatory cascade. TNF-α and IL-6 can inhibit the activity of the insulin signaling pathway by interacting with insulin receptor substrate-1 (IRS-1) in which TNF-α increases the level of the inactivated form of IRS-1 by actuating IRS-1-devitalized kinase and activating the IRS-1 serine residue for phosphorylation (IRS-1 inactivated form). TNF-α can also impede insulin signaling and alter insulin-induced glucose uptake by propelling the PI3K/AKT/mTOR signaling pathway to phosphorylate IRS-1 serine. Meanwhile, IL-6 prevents the interaction between the insulin receptor and IRS-1, which can degrade the phosphorylation of the tyrosine residues of IRS-1 (active form level of IRS-1) or function of the insulin signaling pathway and then block the PI3K/AKT pathway. Both aforementioned cytokines can transform the natural action path of insulin in PCOS patients via diverse pathways, leading to IR, which influences the glucose metabolism of the endometrium and harms receptivity [64]. Another cytokine, lipopolysaccharide (LPS), may enter the blood when the intestinal barrier function is impaired. LPS-binding protein (LBP) binds to CD14 Toll-like receptor-4 complex (TRL-4) on the surface of immune cells and activates downstream signaling pathways, prompting the excitation of the immune system, further interfering with the insulin receptor function and enhancing serum insulin levels. When the intestinal barrier function is impaired, the entry of endotoxin produced by intestinal flora into the blood causes IR, indicating that IR and chronic inflammation are highly correlated and promote each other. This process forms a vicious cycle, thereby disrupting the physiological endocrine and metabolic microenvironments of the endometrium and affecting tolerance [50, 65].

### 3.3 Effect of insulin resistance (IR) on endometrial receptivity in PCOS patients

#### Physiological state

The entire process of embryo implantation and development requires a large amount of energy, mainly involving glucose, its uptake and utilization reflect a fundamental characteristic for the proper differentiation of the endometrium. Consequently, endometrial IR may reduce glucose uptake in endometrial cells and interfere with endometrial receptivity. Under physiologic conditions, insulin initiates a cascade of amplification reactions via signaling molecules after binding to its specific receptor. Two major signaling pathways have been identified. One is the PI3K/AKT pathway, which mainly involves the substance metabolism of cells—that is, insulin tyrosine phosphorylates the insulin receptor by initiating the PI3K/AKT pathway and increases the uptake and utilization of peripheral insulin. The other is the mitogen-activated protein kinase/extracellular signal-regulated kinase pathway, which mainly participates in cell differentiation, proliferation, and apoptosis. Aberrant insulin signaling pathways that regulate glucose metabolism in the endometrium are a powerful mechanism for the pathogenesis of PCOS patients. Owing to the presence of an error in any link of these two pathways, insulin fails to function properly, which can lead to IR.

#### PCOS patients

The main cause of IR in PCOS patients is the inhibition of the insulin signaling molecule transduction pathway due to the phosphorylation of the serine residues of the insulin receptor substrate molecule [102]. Moreover, alterations in certain genetic material may lead to blockage of insulin secretion and cause IR, such as the MTNR1B gene [91]. The pathological process of IR in PCOS patients is suggested to be concerned with the disturbance of intestinal flora. The diversity of α and β in the PCOS group is lower than that in the control group. Consequently, the connection between intestinal epithelial cells is destroyed, leading to accessorial intestinal mucosal permeability. LPS leaks into the humoral circulation via the intestinal mucosa and activates the immune system. Meanwhile, the activation of the immune system can affect the sensitivity and normal function of insulin receptors [28]. In addition, hyperandrogenism impedes the endometrial insulin metabolic pathway and reduces glucose uptake in the basal state and response to insulin stimulation of the endometrium [63], indicating that androgens can spawn partial IR in the endometrium. By interfering with glucose metabolism, its mechanism may hinder the utilization of endometrial glucose, as well as the growth and activity of endometrial cells. Such an obstacle causes endometrial dysplasia, thinning, blockage of decidualization, and damage to endometrial receptivity [68]. The link between the glucocorticoid level and IR is also extremely close. Studies suggest that in contrast with the matched group, the PCOS group showed a reduction in 11β-hydroxysteroid dehydrogenase(11β–HSD2). Simultaneously, the local oxidation of cortisol was reduced, whereas that of cortisone was increased—that is, the secretion of active glucocorticoids in the body is elevated. However, cortisol restrains glucose uptake, AKT phosphorylation, and exocytosis of glucose transporter 4(GLUT4) to the cell membrane, which induce the further occurrence and development of IR [68].

Research indicates that in IVF–ET cycles, no significant differences in oocyte maturation rate and embryo quality exist between PCOS patients with IR and PCOS patients without IR. Nonetheless, the embryo implantation rate and clinical pregnancy are significantly reduced, and the expression of markers related to endometrial receptivity are influenced in the PCOS patients with IR [14]. This observation shows that IR in the PCOS group may be closely associated with defective endometrial receptivity. During endometrial implantation, one of the key hallmark molecules in decidualization is IGFBP-1 [94], at which point the expression of insulin growth factor-1 is markedly increased, whereas that of IGFBP-1 is decreased in PCOS patients relative to those in non-PCOS patients. The population is more likely to experience miscarriage, and the reason may be that IR in PCOS patients reduces IGFBP-1 expression and inhibits the decidualization of endometrial stromal cells, which then hampers embryo implantation [56]. Equally important is the downregulation of PI3K/AKT signal transduction pathway-related molecules in PCOS patients who have IR attributed to insulin damage. Accordingly, the number of GLUT4 transported to the cell membrane was decreased or ectopic. Meanwhile, the transport of GLUT4 vesicles from intracellular deposits to the plasma membrane has a crucial role in maintaining glucose homeostasis, and their exceptional translocation is strongly associated with IR. In this case, the glucose required for endometrial stromal decidualization cannot be transported into the cells. Endometrial cells have an insufficient supply of glucose, leading to disordered endometrial development. Distinctly thinner endometrium than that in normal pregnant women is thus produced, which interferes with endometrial receptivity [45]. In the control group, GLUT4 was strongly positive in endometrial epithelial cells but weakly positive in stromal cells; meanwhile GLUT4 was weakly positive in the endometrial and stromal cells of PCOS patients. After treatment with metformin, GLUT4 levels in PCOS patients returned to the baseline of control levels, indicating that metformin can increase peripheral glucose transport and improve IR by affecting GLUT4 expression in the endometrial cells of PCOS patients. These effects enhance endometrial receptivity in PCOS patients [107]. In vitro studies have confirmed that the adiponectin (APN) signaling pathway is blocked in obese PCOS patients presenting with IR, unlike that in controls; and one of the vital roles of APN is to increase insulin sensitivity. Therefore, the endometrial function is disturbed, and receptivity is reduced in such patients [22].

### Effect of endometrial morphology and thickness on endometrial receptivity in PCOS patients

#### Physiological state

The endometrium can be divided into three different morphologies—A, B, and C—as determined from its images on ultrasound, which appear alternately with a change in sex hormones throughout the menstrual cycle in women. In the proliferative phase, the endometrium gradually thickens with an increase in E. At this time, ultrasound exhibits a high echo between the endometrium and the muscular layer. The central line formed by the two layers of endometrial borders was also high echo, assuming a typical "three-line sign" known as type A. In the late proliferative phase, the endometrium further thickens, showing a uniform moderate echo on ultrasound. The central line known as type B is intermittently unclear. The endometrium entering the secretory phase thickens under the combined action of E and P. It is also filled with abundant glycogen and other nutrients, demonstrating homogeneous hyperechogenicity on ultrasound; however, the central line is difficult to visualize. By this time, the "three-line sign" known as type C disappears. Type A endometrium has higher implantation than types B and C, and the presence of hyperechoic endometrium in the follicular phase predicts adverse pregnancy outcomes [52].

#### PCOS patients

The secretory endometrium of PCOS patients has been proved to mostly present type B or C. The reason for this may be the disturbance of relevant hormone levels after ovulation. Subsequently, focal dense endometrial stroma develops, which ultimately leads to poor endometrial secretion, excessive hyperplasia, or local microenvironment abnormalities. The rate of type-A endometrial stromal dysplasia is markedly lower than that of non-type A (P < 0.05), as observed by optical microscopy, indicating that the enhancement of endometrial echogenicity is mainly associated with stromal dysplasia [108]. The proportion of PCOS patients with type A endometrial morphology, treated using letrozole, is notably higher than that of the control group, improving endometrial receptivity, as well as increasing clinical pregnancy and ongoing pregnancy rates [3, 103].

The female endometrium layer can be split, according to whether it changes with sex hormones, into the basal layer and the functional layer. The functional layer is in the process of dynamic change, and its proliferation and secretion status undertake a key role in the positioning, adhesion, and implantation of fertilized eggs. A trial using transvaginal ultrasound parameters to assess endometrial receptivity in PCOS patients considered that in PCOS patients, endometrial thickness ≤ 7 mm was a risk factor for impaired endometrial receptivity, with an OR of 5.223. The mean endometrial thickness was 7.45 ± 0.38 in PCOS patients with appropriate endometrial receptivity. Under this condition, the RI and PI values of endometrial arteries with this thickness were low, whereas the blood perfusion of the endometrium was high. Consequently, endometrial receptivity was high, which was beneficial to embryo implantation, whereas endometrial thickness in PCOS patients with inferior endometrial receptivity was significantly thin, only 5.96 ± 0.64 (*P* < 0.01) . From the proliferative phase to the secretory phase, under the stimulation of steroidal hormones and the supply of endometrial blood flow, the endometrium gradually thickens, the glands gradually grow, and blood vessels increase and bend. This series of physiological transformations provide rich nutrients and conditions for the establishment of proper endometrial receptivity, which is conducive to the implantation of fertilized eggs. The endometrial thickness measured on the day of hCG injection was clearly higher in the group with clinical pregnancy than in that group without clinical pregnancy, indicating that insufficient endometrial thickness may affect the "dialogue" mechanism between the embryo and the endometrium, thereby reducing the implantation rate and clinical pregnancy rate [16]. Increasing the endometrial thickness within a certain range distinctly improves the implantation rate and cycle pregnancy rate of patients undergoing frozen embryo transfer than those of the control group by 2.24 and 2.32, respectively [19]. The use of certain medications can reverse radiation-induced uterine fibrosis and improves endometrial thickness, consequently refining endometrial receptivity. Conclusively, subjects with poor endometrial receptivity treated with pentoxifylline and tocopherol for 6 months exhibited an increase in endometrial thickness from 4.9 mm to 6.2 mm and improvement in endometrial receptivity, following a significant increase in pregnancy rate [90] (Table 1). Although most scholars suggest that endometrial thickness has a high predictive value for endometrial receptivity, no clear evidence supports this finding, and the study on PCOS patients is insufficient. More clinical studies are needed to confirm the relationship between the two.

**Table 1:** Researches of relationship between endometrial features and endometrial receptivity

**Research**	**Research design**	**Country**	**Inclusion population**	**Sample size**	**Leading indicator**	
Sedigheh Amooee, [87]	Retrospective cross-sectional study	Iran	PCOS infertility patients aged 15–38 years	70	endometrial histology, luteinizing hormone, follicle stimulating hormone, thyroid stimulating hormone, testosterone, prolactin, fasting blood glucose, body mass index ( BMI) and duration of infertility	The histological results of women with PCOS showed that proliferative endometrium accounted for 54.3%, proliferative abnormal endometrium accounted for 17.1%, secretory endometrium accounted for 8.6%, endometrial polyps accounted for 17.1%, and these percentages of UI patients were 28.6%, 0%, 54.3%, and 20%, respectively
Li Wang, 2019 [103]	randomized controlled trial	China	Patients diagnosed with PCOS	160	endometrial thickness, morphology, uterine artery blood flow, subendometrial blood flow, endometrial volume and vascularization index, biochemical pregnancy rate, clinical pregnancy rate, persistent pregnancy rate	After PCOS patients use of letrozole, their endometrial thickness had increased and the morphology was most of type A, which improves endometrial receptivity and improves pregnancy rates
Manal T Al-Obaidi, 2019 [3]	Random blind-free control experiment	Iraq	Infertile PCOS patients	80	Follicle growth on day 12–13, endometrial thickness, Uterine artery resistance index, pulsatility index, contraction / diastolic velocity ratio	Compared with the clomiphene citrate group, the endometrial thickness was significantly higher in the cozole group. The resistance index and pulsatility index of the patients in the cozole group and pregnant women were lower than those in the non-pregnant group
Shaoquan Shi, [93]	case control study	China	PCOS patients resistant to clomiphene citrate	96	Number of growing and mature follicles, serum estrogen, progesterone, endometrial thickness, pregnancy rate, abortion rate	There was no significant difference in the number of mature follicle cycles between HMG group and letrozole group, clinical pregnancy rate and abortion rate. There was no significant difference in endometrial thickness between the two groups on the day of HCG injection; Serum estradiol ( E2) was lower in letrozole group. The incidence of ovarian cysts was lower than HMG group. The incidence of ovarian hyperstimulation syndrome in letrozole group was lower than that in HMG group
Taosa, [96]	case control study	China	PCOS infertility patients with normal uterus and ovary morphology	162	Vaginal ultrasound parameters, endometrial thickness and volume, Uterine artery resistance index, pulsatility index, contraction / diastolic velocity ratio	When endometrial thickness ≤ 7.0 mm, endometrial volume ≤ 2 mL, uterine spiral artery PI ≥ 1.6, RI ≥ 0.72 and S/D ≥ 3.6, PCOS patients have an increased risk of poor endometrial receptivity
Maryam Eftekhar, 2018 [19]	randomized controlled trial	Iran	Patients with poor endometrial receptivity receiving embryo transfer	83	endometrial thickness, chemical pregnancy rates, clinical and ongoing pregnancy rates	Increasing endometrial thickness within a certain range, especially when endometrial thickness is ≥ 7 mm, can significantly increase the implantation rate and cycle pregnancy rate in patients undergoing frozen embryo transfer

The table lists the relevant studies on the relationship between endometrial thickness, morphology and endometrial receptivity in recent years. Their included population was all PCOS patients

## Conclusion

In summary, multiple evaluation indexes of endometrial receptivity in PCOS patients vary from those in normal women of childbearing age. Their endometrial receptivity is closely associated with gene expression, energy metabolism, and endocrine environment. The long-term distinct pathological process in such a population impairs endometrial receptivity, resulting in the blockage of embryo implantation. Despite numerous analyses of the correlative markers of endometrial receptivity in PCOS patients and their mechanism of action, a further study on their clinical relevance is necessary. Thus, addressing these problems in future research and clinical practice bears significance.

## Abbreviations

PCOS: Polycystic ovary syndrome
IR: Insulin resistance
NIH: National Institutes of Health
AES: Androgen Excess Society
LH: Luteinizing hormone
FSH: Follicle-stimulating hormone
E1: Estrone
E2: Estradiol
AMH: Anti-Müllerian hormone
pps: Pinopodes
LIF: Leukemia inhibitory factor
ER: Estrogen receptor
PR: Progesterone receptor
E: Estrogen
P: Progesterone
GnRH-α: Gonadotropin-releasing hormone agonist
NCS: Nucleolar channel system
hCG: Human chorionic gonadotropin
HOXA-10: Homeobox gene-A10
HOXA-11: Homeobox gene-A11
IGFBP-1: Insulin-like growth factor binding protein-1
miRNAs: MicroRNAs
MMPs: Matrix metalloproteinase
IL-6: Interleukin-6
LOD: Laparoscopic ovarian drilling
LGR4: G protein-coupled receptor 4
GnRH: Gonadotropin-releasing hormone
AR: Androgen receptor
T: Testosterone
uNK cells: Uterine natural killer cells
EVT: Extra villous trophoblast
TNF-α: Tumor necrosis factor-α
IRS-1: Insulin receptor substrate-1
LPS: Lipopolysaccharide
LBP: LPS-binding protein
TRL-4: Toll-like receptor 4
11β–HSD2: 11β-Hydroxysteroid dehydrogenase
GLUT4: Glucose transporter 4
APN: Adiponectin
